# Comment on Szczubełek et al. Effectiveness of Crohn’s Disease Exclusion Diet for Induction of Remission in Crohn’s Disease Adult Patients. *Nutrients* 2021, *13*, 4112

**DOI:** 10.3390/nu14091733

**Published:** 2022-04-22

**Authors:** Emilie van Lingen, Sander van der Marel, Jeroen Maljaars, Josbert Keller, Andrea van der Meulen-de Jong

**Affiliations:** 1 Department of Gastroenterology and Hepatology, Leiden University Medical Center, 2333 ZA Leiden, The Netherlands; p.w.j.maljaars@lumc.nl (J.M.); j.keller@haaglandenmc.nl (J.K.); ae.meulen@lumc.nl (A.v.d.M.-d.J.); 2 Department of Gastroenterology and Hepatology, Haaglanden Medical Center, 2512 VA The Hague, The Netherlands; s.van.der.marel@haaglandenmc.nl

We read with interest the manuscript by Szczubelek et al. [1], discussing the Crohn’s disease exclusion diet (CDED) in mild-to-moderate adult CD patients.

The results of this study are encouraging, with a clinical remission rate of 77% at week 6 and up to an 82% sustained remission at 12 weeks. Of note, 88% of patients completed the study, which indicates a high compliance. Similar results were obtained in the recently published study of Yanai et al. [2]. We would like to react based on our recently conducted pilot study, with a similar set up but more disappointing results.

Already early on in the COVID-19 pandemic, the challenge arose among IBD patients and physicians for alternative treatment options to immunosuppressive therapy, even though it was soon recognized that immunosuppressive medication under the pressure of the pandemic still appeared to be safe and effective. To meet this demand, and with the promising results presented by Levine et al. [3], we performed a pilot study of CDED with PEN (Modulen IBD; Nestlé) in CD patients with mild-to-moderate active IBD who actually needed induction therapy, such as prednisolone or biologicals. The study design was similar to the study of Szczubelek et al., with a strict diet in the first 6 weeks and an increase in permitted food products in the following 6 weeks [1]. Moreover, we also included patients who were not treatment-naïve.

From June 2020 until June 2021, 12 CD patients started with CDED in a gastroenterology outpatient center. In the end, 58% of the patients (7/12) did not complete Phase I and II of the study because of an increase in complaints (*n* = 3), losing too much weight (*n* = 2) and a lack of compliance with the strict diet (*n* = 2), despite the undiminished commitment of dieticians and physicians in the outpatient clinic. In contrast to Szczubelek et al., we noticed that 17% (2/12) of patients were losing weight during CDED, which is undesirable during an IBD flare. Nonetheless, five out of twelve patients completed the study, from which three patients noticed clinical improvement at the end of follow up, although in two of these three patients, the calprotectin level still remained high. 

Despite the observations in our small cohort of patients, we concur with Szczubelek et al. that CDED could potentially be a very valuable treatment method for adult CD patients. The focus on the implementation of diet for CD in the future should be adjusted depending on the country of the research, assessed in specified smaller groups and performed as an international study with a uniform protocol for both doctors and dieticians. Such a powerful, randomized controlled trial might give us the tools and answers that we need for the implementation of CDED treatment in the future.

## Data Availability

The dataset generated during and/or analysed during the current study are available from the corresponding author on reasonable request.
